# Effects of *Rhizopus*-*arrhizus*-31-Assisted Pretreatment on the Extraction and Bioactivity of Total Flavonoids from *Hibiscus manihot* L.

**DOI:** 10.3390/molecules29051046

**Published:** 2024-02-28

**Authors:** Xiurong Ju, Tao Chen, Yutao Ding, Dan Yu, Jingyu Zhang, Ruyuan Zhang, Yang Zhang, Xinyu Wang, Tao Xu, Jiayou Li

**Affiliations:** 1 College of Life Sciences and Medicine, Zhejiang Sci-Tech University, Hangzhou 310018, China; jxr115831@163.com (X.J.); xutao1998cn@163.com (T.X.); 2 Suqian Product Quality Supervision and Testing Istitute, Suqian 223800, China; cxinyu888@eyou.com; 3 College of Biological, Chemical Sciences and Engineering, Jiaxing University, Jiaxing 314001, China; 13675730361@163.com (Y.D.); yudan_dy@163.com (D.Y.); m19706120992@163.com (J.Z.); zhangruyuanzry@hotmail.com (R.Z.); zhang5201021yang@126.com (Y.Z.); 19857389093@163.com (X.W.)

**Keywords:** *Rhizopus arrhizus*, *Hibiscus manihot* L., total flavonoids, fermentation, antioxidant activity, UPLC-QTOF-MS, chromatograms

## Abstract

The *Hibiscus manihot* L. (HML) Medic, an edible hibiscus of the Malvaceae family, is abundant with flavonoids. The study investigated how *Rhizopus*-*arrhizus*-31-assisted pretreatment affects the extraction and bioactivity of flavonoids from HML. The fiber structure of the fermented flavonoid sample (RFF) appears looser, more porous, and more disordered than the unfermented flavonoid sample (RUF). RFF demonstrates milder conditions and yields higher extraction rates. According to the Box–Behnken response surface optimization experiment, the optimal conditions for RFF include a material–liquid ratio of 1:41 g/mL, a 2 h extraction time, a 57% ethanol concentration, and an extraction temperature of 800 °C, resulting in a 3.69% extraction yield, which is 39.25% higher than that of RUF. Additionally, RFF exhibits greater activity than RUF in the radical-scavenging system. The IC50 values for DPPH, OH, and ABTS radicals are 83.43 μg/mL and 82.62 μg/mL, 208.38 μg/mL and 175.99 μg/mL, and 108.59 μg/mL and 75.39 μg/mL for RUF and RFF, respectively. UPLC-QTOF-MS analysis of the active components in the HML flavonoid sample revealed significant differences in the chromatograms of RUF and RFF, indicating that biofermentation led to substantial changes in composition and content from HML.

## 1. Introduction

*Hibiscus manihot* L. (HML), also known as vegetable hibiscus, is an annual herbaceous plant belonging to Malvaceae and Abelmoschus Medicus [1]. Its flowers emit a pleasant fragrance and have both medicinal and culinary applications. HML boasts a higher concentration of flavonoids compared to plants such as ginkgo and soybean. Key flavonoids found in HML include rutin, hyperin, isoquercitroside, quercetin 3-*O*-β-d-glucofuranoside, and quercetin 3-*O*-robinobiosid [2,3]. Additionally, it contains various other active compounds such as polysaccharides, organic acids, and trace elements [4,5]. These bioactive compounds exhibit a range of physiological benefits including strong antioxidant properties, anti-pain and anti-inflammatory effects, cholesterol reduction, anti-cancer activity, and immune enhancement [6,7,8,9]. Therefore, the extraction of flavonoids from HML holds significance in pharmaceuticals, food additives, and cosmetics industries.

Various methods can enhance the extraction of Chinese herbal medicine, including chemical and physical techniques, enzymes, and microbial fermentation [10]. Solid-state fermentation, in particular, is a widely utilized fermentation method in food processing, agriculture, biomass energy, bioconversion, and detoxification [11]. This technique offers several advantages: (1) it employs a straightforward process and can utilize diverse materials. Agricultural and other wastes can undergo bioconversion without prior treatment [12]. (2) It is associated with lower production costs and requires minimal equipment investment. (3) The process yields higher-quality and more active fermentation products [13]. (4) Microbial fermentation enhances Chinese herbal medicine, promoting water and energy conservation while reducing waste [14]. Microbial fermentation has therefore emerged as a sustainable, energy-efficient, and effective technique.

In recent years, there has been a growing interest in fermented foods due to their potential to enhance nutrition and health benefits. Early studies suggest that fermentation can elevate the antioxidant levels in food. For instance, fermenting black beans with Bacillus subtilis increases the total flavonoid and phenol content [15]. Aspergillus niger can ferment wheat bran, thereby improving its physical and chemical qualities [16]. Fungal fermentation of bran increases levels of phenols, alkyl resorcinol, and antioxidants. Liu et al. [17] demonstrated that fermenting dandelion with fungi significantly boosts the levels of total flavonoids, which can effectively eliminate harmful free radicals and enhance overall antioxidant capacity. Their study identified 57 different types of flavonoids present after fermentation, compared to none before. Further research on fungi fermenting HML revealed changes in flavonoid content and antioxidant activity, suggesting potential applications in developing and utilizing fermented HML foods. *Rhizopus arrhizus* [18], a filamentous fungus, is widely found in both plants and animals. It is a versatile and adaptable fungus and can convert various carbon and nitrogen sources. Additionally, it exhibits a strong ability to produce acids and enzymes, including lactic acid, ethanol, and amylase [19,20,21,22]. Enzymes, with their high specificity and catalytic efficiency, can break down cell walls, allowing active compounds within the cytoplasm to overcome the dual barriers of cell walls and the extracellular matrix. This results in more complete extraction of active substances, faster reaction rates, and ultimately, higher extraction efficiency.

This study optimized the pretreatment process for *Rhizopus*-*arrhizus*-31-assisted extraction of total flavonoids from HML. Single-factor experiments and Box–Behnken response surface optimization were used to determine the optimal extraction conditions. After purification with the macroporous resin LX-83, the in vitro antioxidant activity of the extracted total flavonoids was assessed using various methods, including DPPH-radical-scavenging, hydroxyl-radical-scavenging, ABTS-radical-scavenging, and Fe^3+^-reducing power assays. The findings of this study are expected to provide a theoretical basis for further optimizing the extraction of total flavonoids from HML.

## 2. Results and Discussion

### 2.1. Changes in the Fiber Structure of HML

**Figure 1:**
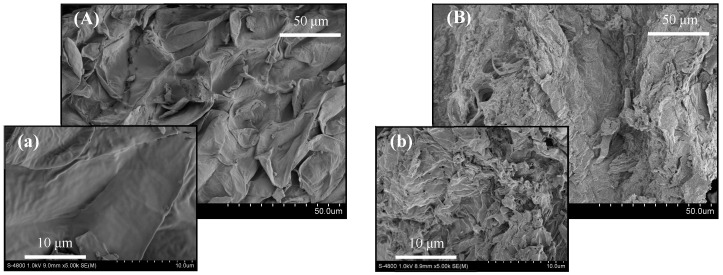
Scanning electron microscope images of HML before and after fermentation. (**A**,**a**) are HML before fermentation, (**B**,**b**) are HML after fermentation; (**A**,**B**) magnification is 1000, (**a**,**b**) magnification is 5000.

Scanning electron microscopy was used to confirm the changes in the structure of HML caused by fermentation. Before fermentation, the surface of the HML cell wall was smooth, flat, and had a solid structure (Figure 1A,a). After fermentation, the cell wall surface became irregular and porous, with honeycomb-like holes, and was covered with inactivated *Rhizopus arrhizus* JHK31 bacteria (Figure 1B,b). Fungal fermentation disrupted the fiber structure, facilitating the release of active substances from the fibers and thereby boosting the extraction of total flavonoids.

Over the course of fermentation, the color of HML gradually transformed from a golden yellow to a light brown, ultimately deepening to a dark brown hue. Initially characterized by a sour scent, the aroma gradually shifted to a characteristically musty one. Similarly, the texture underwent a significant change, starting dry and fluffy and progressively becoming lumpy and sticky. Upon drying, the final texture transformed into a loose, gravel-like consistency.

### 2.2. Effects of Extraction Factors on Total Flavonoid Production

#### 2.2.1. Determination of Solid–Liquid Ratio

We compared the extraction yield of total flavonoids from raw unfermented HML (RUF) and roasted fermented HML (RFF) at different material-to-liquid ratios. Consistently, RFF showed a higher extraction yield than RUF, demonstrating that fermentation pretreatment effectively reduces the required solvent volume. This translates to lower analysis costs and minimal organic solvent residues, making the process more environmentally friendly.

**Figure 2:**
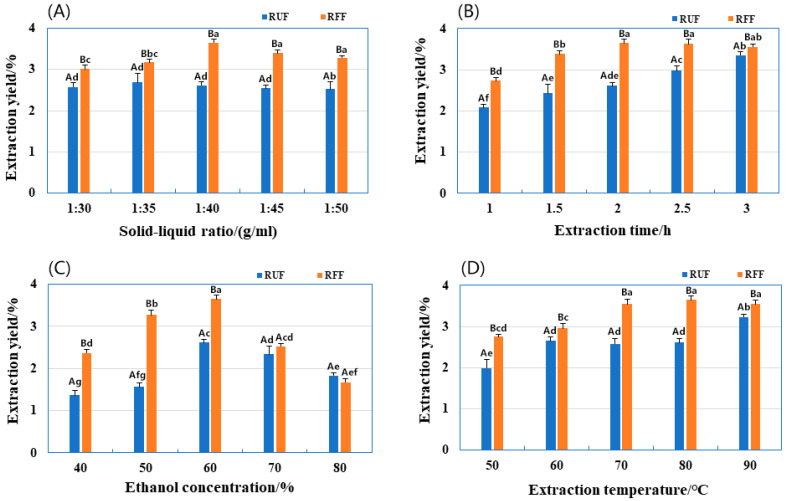
Effects of extraction factors on the total flavonoid extraction yield of RUF and RFF in single-factor experiments. (**A**) Effects of material–liquid ratio; (**B**) Effects of extraction time; (**C**) Effects of ethanol concentration; (**D**) Effects of extraction temperature. Values are expressed as means ± SD (*n* = 3). Different lowercase letters indicate significant differences between RUF and RFF under different extraction conditions (*p* < 0.05); Different uppercase letters indicate significant differences between RUF and RFF under the same extraction conditions (*p* < 0.05).

Figure 2A illustrates how increasing the material-to-liquid ratio of RFF from 1:30 to 1:40 enhances the extraction yield. This is due to two factors: (1) the larger contact area between the sample and solvent allows for better interaction and (2) enhanced cell swelling facilitates the release of total flavonoid content. At a ratio of 1:40, the release and diffusion of total flavonoids reach equilibrium, resulting in a significant 39.85% increase in extraction yield for RFF compared to RFF (3.65%). Increasing the ratio further (beyond 1:40) can lead to a decline in extraction yield due to the co-extraction of impurities with the flavonoids, forming precipitates and hindering the release of pure flavonoids [23].

An analysis of peak extraction yield at 1:40 compared to adjacent ratios of 1:35 and 1:45 revealed a significant 13% difference between the highest and lowest values (*p* < 0.05). This confirms that a material-to-liquid ratio of 1:40 is optimal for subsequent response surface analysis.

#### 2.2.2. Determination of Extraction Time

Figure 2B illustrates the impact of extraction time on the yields of RUF and RFF. Extending the extraction time from 1 h to 1.5 h significantly increased the yield for both samples. However, for RFF, the increase in yield slowed down after 1.5 h, reaching a maximum of 3.65% at 2 h. In contrast, RUF reached its maximum yield (3.35%) only at 3 h. This difference likely reflects the fact that longer extraction times allowed for the release of more total flavonoids from HML, while also extracting other soluble substances. However, these non-flavonoid substances diminished as the extraction time was further extended, resulting in a plateau in the total flavonoid yield [24]. Since fermentation accelerates flavonoid diffusion, extending the extraction time beyond 2 h had little impact on the final yield for RFF. Therefore, 2 h was selected as the optimal extraction time for further analysis using the response surface method.

#### 2.2.3. Determination of Ethanol Concentration

In Figure 2C, we show the extraction yields of RUF and RFF at different concentrations of ethanol. We found that the extraction yields initially increased and then decreased as the ethanol concentration increased, between 40% and 80%. When the ethanol concentration was between 40% and 60% and the water content was high, not all flavonoids were released. However, as the ethanol concentration increased, the solubility of flavonoids also increased, leading to higher extraction yields. At 60% ethanol, RFF had a 1.4 times higher extraction yield than RUF. However, when the ethanol concentration was higher than 60%, it became harder to release larger-polarity flavonoids [25]. Other substances like fats and alcohols posed challenges for separation, reducing the overall flavonoid extraction yield [26]. At this point, there was no significant difference in extraction yields between RUF and RFF. Therefore, we chose 60% ethanol as the central concentration for further experiments.

#### 2.2.4. Determination of Extraction Temperature

Figure 2D shows that as the extraction temperature increased from 50 °C to 90 °C, the extraction yield of RFF remained consistently higher than that of RUF. For RFF, the yield initially increased with temperature, peaking at 3.65% at 80 °C, then decreased. In contrast, RUF reached its maximum yield of 3.23% only at 90 °C. This means RFF achieved a 1.13 higher yield than RUF at its peak. This trend can be explained by the impact of temperature on the diffusion and solubility of flavonoids. Within a certain temperature range, heating accelerates the diffusion and extraction of these compounds, leading to an increase in yield [27]. However, at higher temperatures, flavonoids become more susceptible to degradation, resulting in a decline in yield. Since fermentation allows for lower extraction temperatures while maximizing yield, it offers a more environmentally friendly and energy-conserving approach. Consequently, 80 °C was chosen as the optimal temperature for further analysis using the response surface method.

### 2.3. Response Surface Optimization Analysis

#### 2.3.1. Box–Behnken Response Surface Optimization Experiment Results

According to the results of the single-factor experiments, the material–liquid ratio (A), extraction time (B), ethanol concentration (C), and extraction temperature (D) were selected as evaluation indices for response surface analysis. The results are described below (Table 1 and Table 2).

#### 2.3.2. Parameter Optimization for RFF Extraction

Design-Expert (Version number 13.0.1.0) software was applied to analyze the experimental results using multiple quadratic regression. The regression model was subjected to variance analysis and the results are presented in Table 2. The extraction yield of RFF was predicted by a second-order polynomial.

Y = 3.62 + 0.0808 A + 0.0650 B − 0.2658 C − 0.0217 D + 0.0375 AB − 0.1000 AC − 0.0250 AD − 0.0100 BC +  0.1075 BD − 0.0475 CD − 0.2521 A^2^ − 0.2333 B^2^ − 0.5521 C^2^ − 0.2033 D^2^ (1)

The regression model demonstrated high significance with F = 48.64 and *p* < 0.01. The data fit the model well when the lack of fit *p* = 0.7972 > 0.05, The R^2^ = 0.9813 suggests a strong correlation. Both Adj-R^2^ and pred-R^2^ were reasonably consistent (*p* < 0.05), implying the model can effectively explain most of the variance and accurately predict optimal extraction conditions for RFF. 

The influence of various factors on RFF extraction yield varied significantly, as indicated by the F-values. The order of decreasing impact was: ethanol concentration (C) > material–liquid ratio (A) > extraction time (B) > extraction temperature (D). Notably, temperature (D), and several interaction terms AB, AD, BC, and CD, exhibited no significant effects (*p* > 0.05). Conversely, the interaction term AC showed a significant influence (*p* < 0.05). Factors A, B, C, the interaction term BD, and secondary terms all possessed highly significant effects (*p* < 0.01). These findings suggest non-linear relationships between factors and response, highlighting the value of the regression equation in identifying optimal extraction conditions.

#### 2.3.3. Response Surface Analysis and Process Optimization

To understand how the four factors (ethanol concentration, material–liquid ratio, extraction time, and extraction temperature) and their interactions affect RFF extraction yield, we analyzed contour plots. The steepness of the response surface, reflected by the contour shape and slope, indicates the magnitude of the impact on yield. As extraction conditions change, a greater sensitivity between factors, shown by steeper slopes or more eccentric ellipses in the contour shape, signifies a stronger interaction. Conversely, circular contours represent weaker interactions [28].

**Figure 3:**
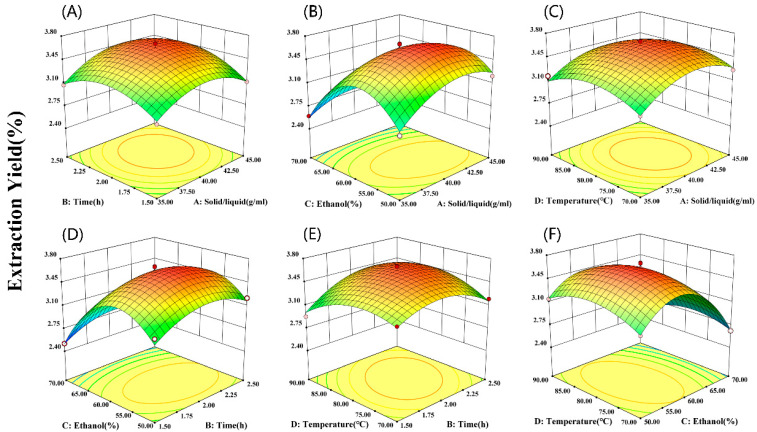
The 3D response surface plots showing the effect of different interactions of factors on total flavonoid extraction yield of RFF, respectively. (**A**) Solid/liquid and extraction time; (**B**) solid/liquid and ethanol concentration; (**C**) solid/liquid and extraction temperature; (**D**) extraction time and ethanol concentration; (**E**) extraction time and extraction temperature; (**F**) ethanol concentration and extraction temperature.

The interaction between the feed–liquid ratio and extraction time displayed a parabolic trend (Figure 3A). The RFF extraction yield increased initially with both factors, reaching a maximum, and then declined. The interaction surfaces between the material–liquid ratio and ethanol concentration suggested a significant effect on the extraction yield. However, ethanol concentration had a more pronounced impact compared to the material–liquid ratio (Figure 3B). There was an interaction between material–liquid ratio and extraction temperature. Similar to the previous interaction, the extraction yield initially increased with both parameters before decreasing (Figure 3C). The interaction between extraction time and ethanol concentration displayed a pronounced elliptical pattern (Figure 3D). The optimal ethanol concentration ranged between 55% and 60%, and the plot suggests a relationship between these factors, potentially influenced by the material’s cellular structure. Extraction time and temperature significantly affected the extraction yield, displaying a parabolic trend. Their interaction was also significant, with the yield initially rising and then falling with increasing extraction time and temperature (Figure 3E). By analyzing the contour plots, we determined the optimal ethanol concentration to be around 60% and the ideal extraction temperature to be 80 °C (Figure 3F).

The regression model predicted optimal RFF extraction conditions as material–liquid ratio 1:41.122 (g/mL), extraction time 2.082 h, ethanol concentration 57.373%, and extraction temperature 80.062 °C. Under these parameters, the anticipated RFF value was 3.672%. For practical purposes, the optimal conditions were adjusted to a material–liquid ratio of 1:41 (g/mL), extraction time of 2 h, ethanol concentration of 57%, and extraction temperature of 80 °C. Under the adjusted conditions, three concurrent validation tests resulted in an actual RFF extraction yield of 3.69%, with a relative error of 0.8%, closely matching the prediction. Compared to RUF, the *Rhizopus*-*arrhizus*-assisted pretreatment of HML resulted in significant benefits: reduced extraction time, lower temperature, less solvent, and a notably higher RFF extraction yield. This confirms the effectiveness of response surface analysis in optimizing the extraction process.

### 2.4. Comparison of Antioxidant Activity of RFF and RUF

Research on total flavonoid extraction mainly focuses on their ability to scavenge and reduce free radicals due to their antioxidant capacity. This ability stems from the reaction between their phenolic hydroxyl groups and free radicals, which results in the formation of stable semiquinone structures, effectively inhibiting the chain reactions that free radicals propagate. Considering that enzymatic activity can influence the antioxidant activity of flavonoids, we assessed the activity of RUF and RFF using multiple methods during fermentation.

**Figure 4:**
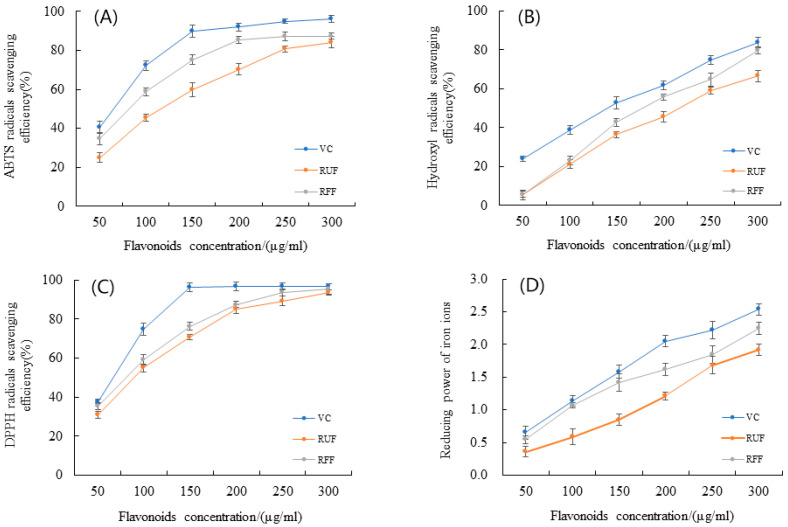
Effects of RFF on radical-scavenging efficiency in vitro antioxidant experiments. (**A**) Effects on ABTS radicals; (**B**) effects on hydroxyl radicals; (**C**) effects on DPPH radicals; (**D**) effects of reducing power on iron ions. Values are expressed as means ± SD (*n* = 3).

This study revealed that both RUF and RFF exhibited significant scavenging and reducing abilities against DPPH, OH, ABTS radicals, and Fe^3+^. A notable positive correlation existed between the concentration of total flavonoid extract and these four indicators. When the concentration of total flavonoids was 300 μg/mL, the scavenging yields for RUF were 93.70% (DPPH), 66.51% (OH), and 84.24% (ABTS), whereas for RFF they were 95.45% (DPPH), 79.63% (OH), and 87.33% (ABTS) (Figure 4). This clearly shows that RFF significantly outperformed RUF in scavenging these radicals, with its antioxidant capacity for DPPH and ABTS approaching that of vitamin C. The IC50 values of RUF and RFF for DPPH, OH, and ABTS radicals were 83.43 μg/mL and 82.62 μg/mL, 208.38 μg/mL and 175.99 μg/mL, and 108.59 μg/mL and 75.39 μg/mL, respectively. These results confirm the significantly superior antioxidant capacity of RFF compared to RUF. Microbial pretreatment can potentially alter the original components of herbal medicines. Further research is needed to understand the specific transformation pathways involved and their impact on the biological activity of these components.

### 2.5. UPLC-QTOF-MS Analysis of RUF and RFF

#### 2.5.1. UPLC-QTOF-MS Analysis of RUF and RFF Chemical Composition

Both RUF and RFF chromatograms exhibited well-separated peaks, indicating satisfactory resolution for various components. This good separation allows for an effective comparison of changes in these components between RUF and RFF.

**Figure 5:**
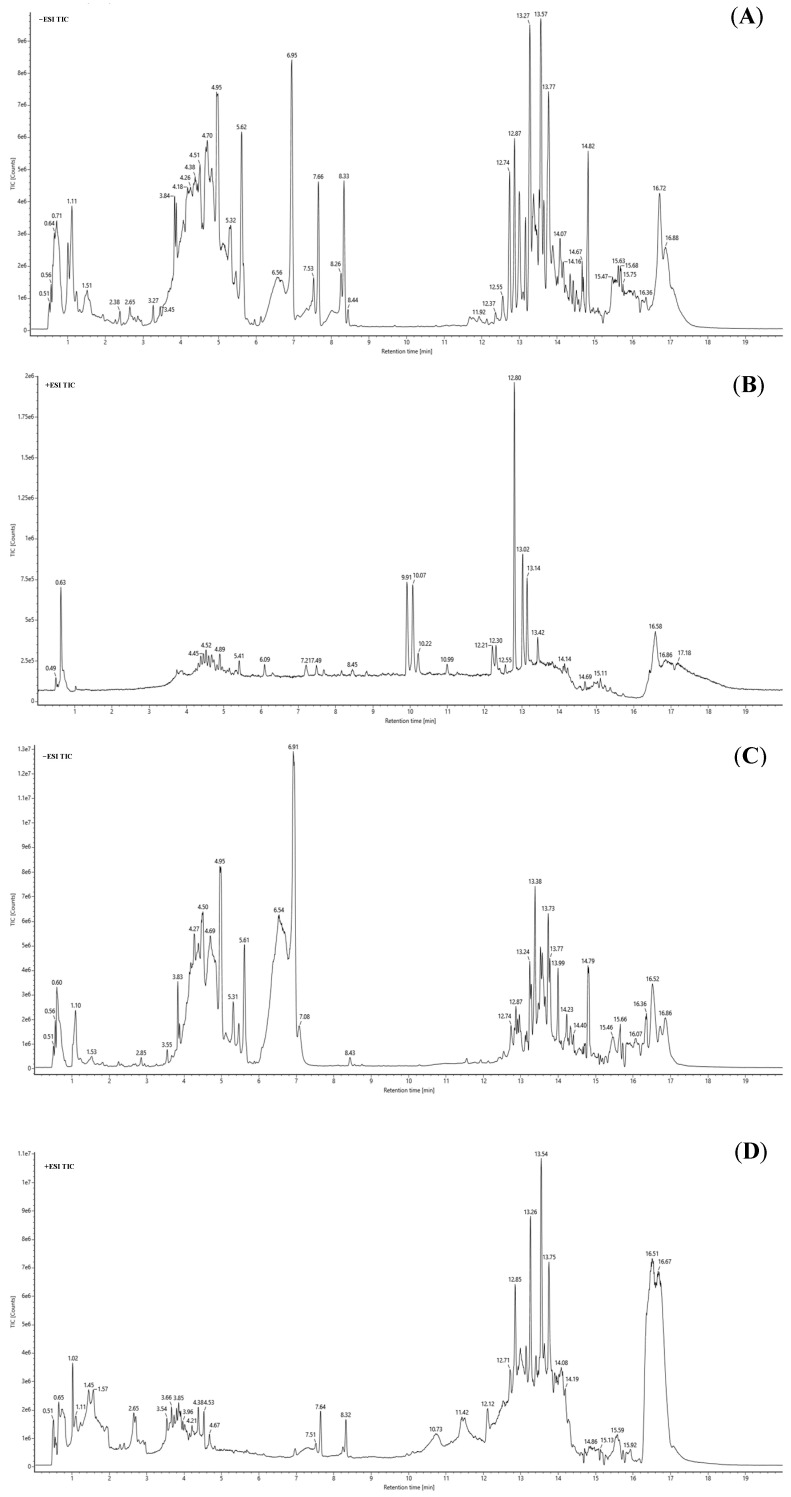
Total ion chromatograms of HML extracts by UPLC-QTOF-MS. (**A**) RUF negative ion mode. (**B**) RUF positive ion mode. (**C**) RFF negative ion mode. (**D**) RFF positive ion mode.

Using the chromatography–mass spectrometry conditions described in Section 3.12, we analyzed and interpreted the compositions of RUF and RFF with the UPLC-QTOF-MS method. We acquired total ion chromatograms in both positive and negative ion modes, as presented in Figure 5. Significant differences were observed in the chromatograms of the two samples, indicating that the flavonoid composition changed substantially during fermentation. By analyzing primary and secondary mass spectrometry data, and comparing them with the PubMed database and Malvaceae family literature, we identified 63 and 87 compounds in RUF and RFF, respectively, as listed in Table 3 and Table 4.

**Figure 6:**
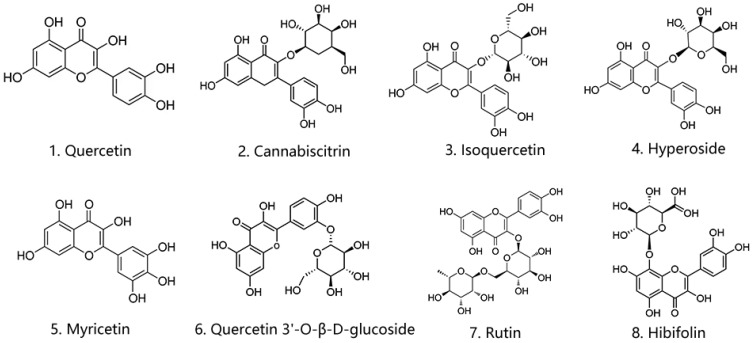
Chemical structures of eight target flavonoids. 1. Quercetin; 2. Cannabiscitrin; 3. Isoquercetin; 4. Hyperoside; 5. Myricetin; 6. Quercetin 3′-*O*-β-d-glucoside; 7. Rutin; 8. Hibifolin.

Analysis showed 52 common peaks between RUF and RFF. Fermentation led to a decrease in the content percentage of 24 substances. The three most significant decreases were observed in hyperoside (12.26%), myricetin 3-*O*-β-d-glucoside (5.79%), and quercetin 3′-*O*-β-d-glucoside (4.82%). Conversely, 28 substances increased in content percentage after fermentation, with the most significant increases seen in quercetin (13.61%), hibifolin (12.26%), and rutin (2.64%). A comparison shows that flavonoids form a significant cluster in the tables. Among them, the eight most abundant compounds in RUF were hibifolin (23.17%), hyperoside (16.57%), isoquercetin (11.61%), quercetin (10.17%), myricetin (8.09%), quercetin 3′-*O*-β-d-glucoside (7.76%), myricetin 3-*O*-β-d-glucoside (6.98%), and cannabiscitrin (5.15%). The eight most abundant compounds in RUF were hibifolin (31.06%), quercetin (23.79%), isoquercetin (12.86%), myricetin (7.43%), rutin (6.81%), hyperoside (4.31%), quercetin 3′-*O*-β-d-glucoside (2.94%), and leeaoside (2.50%). Figure 6 illustrates the chemical structures of quercetin, cannabiscitrin, isoquercetin, hyperoside, myricetin, quercetin 3′-*O*-β-d-glucoside, rutin, and hibifolin.

#### 2.5.2. Analysis of Metabolomic and Enzymes

Preliminary metabolomic analysis showed that the CAMKK2 pathway, vital in quercetin metabolism, significantly impacts biological processes such as cardiac contraction, neurotransmitter release, and cell apoptosis. Notably, this pathway is also associated with the development of neurodegenerative diseases, cancer, and inflammation [29]. Additionally, the AMPK signaling pathway in humans is closely related to various diseases, including diabetes, obesity, and cardiovascular disorders [30]. Digestion and absorption pathways play a crucial role in neural functions, hormonal actions, feeding behaviors, and intestinal microbial activities [31].

Plant enzymes are involved in the biosynthetic pathway of flavonoids [32]. Our review of the literature and subsequent analysis identified key enzymes crucial in the biosynthetic pathway of hibifolin, particularly glucuronosyltransferase [33]. This enzyme either forms or cleaves glycosidic bonds, linking glucuronic acid to hibifolin. This process results in the production of quercetin-8-O-glucuronide and free glucuronic acid. Additionally, glycosidases and glucuronidases contribute to hibifolin metabolism. Glycosidases hydrolyze (break down) glycosidic bonds in hibifolin, releasing quercetin-8-O-glucuronide and other metabolites. Glucuronidases target glucuronic acid, aiding in its metabolism and transformation [34]. 

## 3. Materials and Methods

### 3.1. Materials

*Hibiscus manihot* L. was provided and identified by Yulin Xinbang Pharmaceutical Co., Ltd. (Yulin, China). It was dried in a tray at 65 °C, destemmed and crushed (0.42 mm), and stored at 4 °C for further use.

The fermentation strain was *Rhizopus arrhizus* JHK31. We obtained it through pressure screening. It is stored in the China Center for Type Culture Collection under the deposit number CCTCC M 2023722.

Reagents including rutin (P98%), vitamin C (P99%), aluminum nitrate nonahydrate (P99%), DPPH (P96%), iron trichloride hexahydrate (P99%), trichloroacetic acid (P99%), dibasic sodium phosphate (P99%), and sodium dihydrogen phosphate (P99%) were from Shanghai Macklin Biochemical Co., Ltd. (Shanghai, China). Shanghai Aladdin Biochemical Technology Co., Ltd. (Shanghai, China) provided other chemicals such as ferrous sulfate (P98%), green vitriol (P99%), sodium hydroxide (P96%), Tris (P99.8%), and salicylic acid (P99%). Shanghai yuanye Bio-Technology Co., Ltd. (Shanghai, China) provided hyperoside (P98%), isoquercitrin (P98%), myricetin (P98%), and quercetin (P98%). Other chemicals such as sodium nitrite (P99%), potassium ferricyanide (P99.5%), and pyrogallol (P99%) were analytically pure. Acetonitrile and methanol were of chromatographic grades. The resin used was LX-83 macroporous resin from Sunresin New Materials Co., Ltd. (Xi’an, China). We used ultrapure water in all experiments.

### 3.2. Instruments

High-pressure sterilizer LDZH-150KBS, Shenan Medical Device Factory (Shanghai, China). Digital display thermostat water bath WB-6, MAIKENUO Instrument Co., Ltd. (Changzhou, China). Constant-temperature incubator LRH-150, Sapeen Scientific Instrument Co., Ltd. (Shanghai, China). Drying cabinet DGG-9150G, Senxin Experimental Instrument Co., Ltd. (Shanghai, China). Ultraviolet–visible spectrophotometer UV-1100, Mepada Instrument Co., Ltd. (Shanghai, China). Pipettor YE209AT0043015, DLAB Scientific Co.Ltd. (Beijing, China). Crusher FE220, Zhongxing weiye Instrument Co., Ltd. (Beijing, China). Circulating water vacuum pumps SHZ-DIII, Yarong biochemical instrument factory (Shanghai, China). High-speed centrifuge M18G, Magal Technology Instrument Co., Ltd. (Shanghai, China). Vacuum freeze dryer LGJ-10, Songyuan Huaxing Technology Development Co., Ltd. (Beijing, China).

### 3.3. Preparation of Spore Suspension

We grew the *Rhizopus arrhizus* JHK31 by putting it on a PDA solid medium. It was then incubated at 28 °C for 72 h. To collect the spores of *Rhizopus arrhizus*, sterile saline was poured into the plate, and the spores were scraped with an inoculating ring into a 50 mL centrifuge tube. The tube was then vortexed and shaken. To prepare a spore mixture, we filtered the solution with a lens wipe and cotton to dislodge the mycelium. We used a hematological counting plate to determine the spore concentration and adjusted it to 1 × 10^8^ cfu/mL for fermentation.

### 3.4. Process Flow of Total Flavonoid Preparation

HML was processed through the following steps: destemming, drying, crushing and sieving (40 mesh), sterilization, solid-state fermentation with *Rhizopus arrhizus* JHK31, ethanol reflux extraction, filtration and concentration to a constant volume, total flavonoid content determination, rotary evaporation and concentration, freeze drying, and purification using a macroporous resin. Finally, antioxidant activity was determined.

A fermented total flavonoid sample (RFF) was obtained using ethanol reflux extraction from the fermentation-treated HML. In contrast, unfermented HML was directly subjected to ethanol reflux extraction, resulting in an unfermented total flavonoid sample (RUF).

### 3.5. Preparation of Fermentation Substrate of HML

We used HML from the same batch and sterilized it for use as the experimental material. We took 10 g of HML and put it into 10 mL of sterile water. Then, we added 7.0% spore suspension of *Rhizopus arrhizus* JHK31, 0.2% ammonium sulfate, and 2.0% glucose. The mixture was then adjusted to an initial pH of 6 using a phosphate buffer. The mixture was fermented for 60 h at 28 °C. After fermentation, we spread the samples on a tray and dried them at 65 °C for 24 h. We crushed the samples and sifted them through a 40-mesh screen to acquire HML fermentation substrate. We stored the substrate in a sealed container at 4 °C.

### 3.6. Electron Microscopy Observation of HML Fibers

The powder of HML was evenly adhered to a sample stage equipped with double-sided adhesive tape. Observations and photography were conducted under an electron microscope. The microscope used 15.0 kV of power and magnifications of 1000× and 5000×.

### 3.7. Determination of the Extraction Yield of Total Flavonoid Content

#### 3.7.1. Standard Curve

We used the method from the 2020 China Pharmacopoeia to measure flavonoid content in HML. We plotted the standard curve by using rutin concentration (C, mg/mL) as the *x*-axis and absorbance (A) as the *y*-axis. The rutin standard curve linear regression equation is y = 9.17143 x + 0.0052 (R^2^ = 0.9999).

#### 3.7.2. Extraction of Total Flavonoids and Calculation of Extraction Yield

We extracted them with specific conditions. These conditions include the material–liquid ratio, ethanol concentration, extraction time, and temperature. We spun the mixture at 10,000 r/min for 10 min. The collected liquid was the total flavonoid extract. We transferred 3 mL of the extract to a 25 mL flask and measured its absorbance. The measurement was repeated three times. We used a specific formula to calculate the extraction yield of the total flavonoid content.

(2) $$Y=\frac{C\times V}{m\times 1000}\times 100\%$$

where: Y is the extraction yield of RUF and RFF (%); C is the total flavonoid concentration (mg/mL); V is the volume at the point of determination (mL); m is the weight of the sample (g).

### 3.8. Single-Factor Experiments on the Extraction Yield of RUF and RFF

Single-factor experiments were conducted to evaluate the influence of variables such as material-to-liquid ratio, ethanol concentration, extraction time, and temperature on the extraction yield of RUF and RFF. This assessment aims to define suitable parameter ranges and optimize the extraction process further.

An extraction process was conducted under the following conditions: 1 g sample of HML mixed with a certain amount of ethanol, 2 h extraction time, 80 °C temperature. The study aimed to assess the influence of various material–liquid ratios (1:30, 1:35, 1:40, 1:45, 1:50 (g/mL)) on the extraction efficiency of RUF and RFF. With a fixed material–liquid ratio of 1: 40, extraction time of 2.0 h, and extraction temperature of 80 °C, subsequent experiments were conducted to determine the extraction efficiency under varying ethanol concentrations (40, 50, 60, 70, 80 (%)). A fixed ethanol concentration of 60%, material–liquid ratio of 1:40, extraction temperature of 80 °C, and neutral pH were used to explore the effect of the extraction time (1.0, 1.5, 2.0, 2.5, 3.0 (h)) on the extraction yield of RUF and RFF. A fixed ethanol concentration of 60%, extraction time of 2 h, and material–liquid ratio of 1:40 were used to investigate the effect of the extraction temperature (50, 60, 70, 80, 90 (°C)) on the extraction yield of RUF and RFF.

### 3.9. Box–Behnken Response Surface Optimization Experiment Design for RFF

From the single-factor experiments results, we used the extraction yield of RFF as the response, and material–liquid ratio (A), extraction time (B), ethanol concentration (C), and maceration temperature (D) as the independent variables. A response surface analysis experiment with four factors and three levels had 29 experimental points (Table 5). We utilized the Box–Behnken model and the Design-Expert (Version number 13.0.1.0) software to determine the optimization conditions for total flavonoid extraction.

### 3.10. Purification of RUF and RFF with Macroporous Resin

First, 50 g of the pretreated LX-83 macroporous resin and 10 g of the sample (ultrapure water as the solvent) were weighed into a 250 mL stoppered triangular flask and agitated on a shaking bed at 28 °C for 24 h. The macroporous resin was filtered and transferred to a 250 mL triangular flask after complete adsorption. Then, 100 mL of 70% ethanol was added to the flask and desorbed for 24 h under the same conditions. The mixture was concentrated using rotary evaporation and subsequently freeze-dried to obtain the purified product.

### 3.11. In Vitro Antioxidant of RUF and RFF

#### 3.11.1. Determination of DPPH-Radical-Scavenging Efficiency

The method was adapted from Zhang et al. [35] with modifications to test total flavonoid content at six different levels (50, 100, 150, 200, 250, 300 (μg/mL)). We compared them to a vitamin C solution of the same amount. We mixed 0.3 mL of the sample with 5 mL of 0.1 mM DPPH solution in a test tube and kept it in the dark for 30 min. The absorbance was then measured at 517 nm with 95% ethanol as a reference and absorbance was recorded as $A_{i}$. For another setup, we mixed 0.3 mL of the sample with 5 mL of 95% ethanol and recorded the absorbance as $A_{j}$. Additionally, a control was set up by replacing the sample with 0.2 mL of 95% ethanol and mixing it with 5 mL of DPPH solution, and absorbance was recorded as $A_{0}$. The formula for calculating the DPPH-radical-scavenging yield was as follows:

(3) $$Y=\left(1-\frac{A_{i}-A_{j}}{A_{0}}\right)\times 100\%$$

#### 3.11.2. Determination of Hydroxyl-Radical-Scavenging Efficiency

The method was adapted with modifications from Zhang et al. [36]. To assess the scavenging activity of RFF and RUF, 1 mL of 3 mM H_2_O_2_ solution, 1 mL of 3 mM FeSO_2_ solution, and 0.5 mL of the total flavonoid (50, 100, 150, 200, 250, 300 (μg/mL)) extract were mixed. Then, 1 mL of 3 mM C_7_H_6_O_3_ ethanol solution was added. After thorough shaking, the mixture was incubated in a 37 °C water bath for 15 min. Distilled water was used as a reference. The absorbance at 510 nm was measured and recorded as $A_{X}$. The process was repeated, but H_2_O_2_ was replaced with distilled water and the absorbance was recorded as $A_{X0}$. Additionally, a control was prepared by measuring the absorbance without H_2_O_2_ or flavonoids, recorded as $A_{0}$. An equal concentration of vitamin C was used as a positive control. The scavenging effect of RFF and RUF was expressed as C and calculated using the following equation.

(4) $$C=\frac{A_{0}-\left(A_{X}-A_{X0}\right)}{A_{0}}\times 100\%$$

#### 3.11.3. Determination of ABTS-Radical-Scavenging Efficiency

This method, adapted from Tao et al. [37] with modifications. An ABTS stock solution was prepared by mixing 7 mM ABTS solution and 2.45 mM K_2_S_2_O_8_ solution at a 1:1 (*v*/*v*) ratio and storing it in the dark for 12–16 h. This stock was then diluted with ethanol to an approximate 1:46 (*v*/*v*) ratio, resulting in an absorbance of about 0.7 ± 0.2 at 734 nm. This diluted solution served as the ABTS working solution. For each test, 100 μL of different sample concentrations (50, 100, 150, 200, 250, 300 μg/mL) was added to 8 mL of the ABTS working solution and incubated in the dark for 6 min. Distilled water was used as a reference, and the absorbance at 734 nm was measured and recorded as $A_{1}$. A control was prepared by replacing the sample with ethanol, and the absorbance was recorded as $A_{0}$. Additionally, the absorbance of ethanol alone (replacing the ABTS working solution) was recorded as $A_{2}$. An equal concentration of vitamin C served as the positive control. The scavenging yield of ABTS radicals of RFF and RUF was expressed as E and calculated as follows:

(5) $$E=1-\frac{A_{1}-A_{2}}{A_{0}}\times 100\%$$

#### 3.11.4. Determination of Reducing Power on Iron Ions

The method was adapted from Raza et al. [38] with modifications. Briefly, 1 mL of sample (50, 100, 150, 200, 250, 300 (μg/mL)), 2.5 mL of pH 6.6 phosphate buffer solution (PBS), and 2.5 mL of 1.0% potassium ferricyanide solution were mixed in a cuvette. Following incubation at 50 °C for 20 min, 2.5 mL of 10% trichloroacetic acid (TCA) solution was added to stop the reaction. The mixture was then centrifuged at 3000 rpm for 10 min. Next, 2.5 mL of the supernatant was combined with 2.5 mL of ultrapure water and 0.5 mL of 0.1% ferric chloride solution. After thorough mixing and a 10 min incubation, the absorbance at 700 nm was measured. Vitamin C served as the positive control and a blank without any sample was used for reference.

### 3.12. UPLC-QTOF-MS Analysis of Changes in Flavonoid Components of RUF and RFF

Extraction conditions: 0.1 g of fermented and unfermented powder of HML, 3 mL of methanol (chromatographically pure), extraction with ultrasound (power 200 W, frequency 53 kHz) for 45 min, passage through 0.22 μm microporous filter membrane. Chromatographic conditions: a combination of ACQUITY UPLCTM I-Class and XevoG3XS QTOF, Waters BEH T3 1.8 m chromatographic column (2.1 mm × 150 mm), column temperature of 40 °C. Mobile phase A = 0.1% formic acid solution, B = acetonitrile; gradient program: 0~1 min, 100%→97% A; 1~2 min, 97%→94% A; 2~2.5 min, 94%→88% A; 2.5~11 min, 88%→75% A; 11~13 min, 75%→50% A; 13~15 min, 50%→0% A; 15~17 min, 0% A; 17~20 min, 0%→97% A. Flow velocity 0.4 mL/min, detection wavelength 350 nm, injection volume 10 μL. Mass spectrometry conditions: electrospray ionization (ESI) ion source; a mass range of 50–1200 Da; ionization mode as ESI (+/−) collecting MSE; capillary voltage of 0.5 kV; ion source temperature of 100 °C; desolvation temperature of 280 °C; desolvation gas flow rate of 800 L/h; cone voltage of 40 V; collision energy of low energy 4 V and high energy 15–60 V; data-processing software is UNIFI 1.9.2.

### 3.13. Statistical Analysis

The experimental results were analyzed using Design-Expert (Version number 13.0.1.0) software and Adobe Photoshop CC 2018 (Adobe Photoshop (hjhvfh.top)). All experiments were conducted in triplicate unless otherwise noted.

## 4. Conclusions

This study employed *Rhizopus arrhizus* JHK3 fermentation to extract total flavonoids from HML. During fermentation, these microorganisms secrete powerful enzymes that break down complex molecules like cellulose and pectin in the plant cell wall, facilitating easier extraction of total flavonoids. This enzymatic breakdown reduces obstacles during solvent extraction, leading to faster, more efficient extraction and potentially stronger antioxidant activity in the final product. Optimal extraction conditions were determined through single-factor and response surface experiments, yielding a material–liquid ratio of 1:41 (g/mL), extraction time of 2 h, ethanol concentration of 57%, and extraction temperature of 80 °C. Under these conditions, the extraction yield of RFF (fermented total flavonoids) reached 3.69%. Additionally, both RUF and RFF exhibited significant scavenging abilities for DPPH, OH, and ABTS free radicals. They also displayed reducing power against ferric ions, with four antioxidant capacity indices of RFF surpassing RUF.

Using UPLC-QTOF-MS, specific chromatographic peaks present in the samples before and after fermentation were identified, leading to the determination of 63 and 87 corresponding compounds, respectively. Notably, a significant cluster of flavonoids was observed in both sets of compounds. Among these, 52 peaks remained unchanged, while 24 decreased and 28 increased in intensity after fermentation. This suggests that fermentation transformed certain flavonoids into compounds with potentially higher antioxidant activity. The pathways involved in quercetin metabolism include the CAMKK2 pathway, the AMPK signaling pathway—Homo sapiens (human), and digestion and absorption. Enzymes involved in hibifolin metabolism include glucuronosyltransferase, glycosidases, and glucuronidases.

This study suggests that the growth metabolism of *Rhizopus arrhizus* might convert and utilize flavonoid components, potentially generating new substances with enhanced antioxidant activity in vitro. This hypothesis aligns with findings from similar research conducted by other scholars. For example, a study on the biotransformation of filamentous fungi showed that Trichoderma harzianum NJ01 could convert puerarin to 3′-hydroxypuerarin under optimal conditions. Notably, 3′-hydroxypuerarin exhibited 20 times greater DPPH-radical-scavenging activity and 1.3 times higher solubility compared to puerarin [39]. Additionally, Yuri Lee et al. reported the conversion of flavonoid glycosides to flavonols (quercetin and kaempferol) in silkworm thorn leaves due to Lactobacillus fermentation, leading to a roughly 20% increase in radical-scavenging activity [40]. These findings provide valuable insights for optimizing RFF extraction and related research.

## Figures and Tables

**Table 1:** Experimental design and results of response surface analysis for the RFF extraction process.

Run	A Material–Liquid Ratio (g/mL)	B Extraction Time (h)	C Ethanol Concentration (%)	D Extraction Temperature (°C)	Y Total Flavonoid Extraction Yield (%)
1	−1	−1	0	0	2.99
2	1	−1	0	0	3.11
3	−1	1	0	0	3.07
4	1	1	0	0	3.34
5	0	0	−1	−1	3.10
6	0	0	1	−1	2.66
7	0	0	−1	1	3.15
8	0	0	1	1	2.52
9	−1	0	0	−1	3.08
10	1	0	0	−1	3.26
11	−1	0	0	1	3.17
12	1	0	0	1	3.25
13	0	−1	−1	0	3.11
14	0	1	−1	0	3.22
15	0	−1	1	0	2.52
16	0	1	1	0	2.59
17	−1	0	−1	0	2.85
18	1	0	−1	0	3.21
19	−1	0	1	0	2.60
20	1	0	1	0	2.56
21	0	−1	0	−1	3.27
22	0	1	0	−1	3.20
23	0	−1	0	1	2.93
24	0	1	0	1	3.29
25	0	0	0	0	3.80
26	0	0	0	0	3.51
27	0	0	0	0	3.61
28	0	0	0	0	3.69
29	0	0	0	0	3.68

**Table 2:** ANOVA for the response surface model.

Source	Sum of Squares	df	Mean Square	F-Value	*p*-Value Prop > F	Salience
Model	3.04	14	0.2171	48.64	<0.0001	**
A	0.0784	1	0.0784	17.56	0.0011	**
B	0.0507	1	0.0507	11.36	0.0050	**
C	0.848	1	0.8480	189.96	<0.0001	**
D	5.6 × 10^−3^	1	5.6 × 10^−3^	1.26	0.2816	
AB	5.6 × 10^−3^	1	5.6 × 10^−3^	1.26	0.2819	
AC	0.04	1	0.04	8.96	0.0104	*
AD	2.5 × 10^−3^	1	2.5 × 10^−3^	0.56	0.4676	
BC	4 × 10^−4^	1	4 × 10−4	0.0896	0.7694	
BD	0.0462	1	0.0462	10.35	0.0067	**
CD	9 × 10^−3^	1	9 × 10^−3^	2.02	0.1786	
A2	0.3813	1	0.3813	85.41	<0.0001	**
B2	0.3267	1	0.3267	73.18	<0.0001	**
C2	1.83	1	1.83	409.66	<0.0001	**
D2	0.2481	1	0.2481	55.57	<0.0001	**
Residual	0.0580	13	4.5 × 10^−3^			
Lack of fit	0.0374	10	3.7 × 10^−3^	0.5421	0.7972	
Pure error	0.0207	3	6.9 × 10^−4^			
Cor total	3.10	27				
R^2^	0.9813					
Adj-R^2^	0.9611					

Note: “**” is *p* < 0.01, indicating that the effect is highly significant, and “*” is *p* < 0.05, indicating that the effect is significant.

**Table 3:** Identified chemical constituents from RUF by UPLC-QTOF-MS in positive and negative ion mode.

No.	RT/min	Precursor Ion	Meas.*m/z*	*m/z*	Error/ppm	Formula	Fragments	Identification	Response
1	0.667	[M−H]^−^	266.0891	266.0887	1.5	C_10_H_13_N_5_O_4_	217, 215, 179, 101	Adenosine	4336
2	1.232	[M−H]^−^	610.1539	610.1524	2.5	C_27_H_31_O_16_	471, 470, 443, 308, 279, 133, 128	Enocyanin	124
3	1.769	[M−H]^−^	492.136	492.1354	1.2	C_23_H_28_Cl-N_3_O_5_S	424, 358, 334, 213	Glyburide	239
4	3.553	[M−H]^−^	153.0187	153.0186	0.7	C_7_H_6_O_4_	—	Protocatechuic acid	8653
5	3.692	[M−H]^−^	369.0821	369.0816	1.4	C_16_H_18_O_10_	247, 232, 216, 201, 191	Fraxin	893
6	3.718	[M−H]^−^	677.1536	677.1497	5.8	C_34_H_30_O_15_	611, 477, 305, 189, 146, 144	3,4,5-Tricaffeoylquinic acid	211
7	3.740	[M−H]^−^	299.0765	299.0752	4.3	C_13_H_16_O_8_	196, 146, 93	Salicylic acid-β-d-glucoside	36,072
8	3.795	[M−H]^−^	479.0794	479.0819	−5.2	C_21_H_20_O_13_	447, 425, 333, 300, 175	Myricetin-3-*O*-β-d-glucoside	1,029,648
9	3.796	[M−H]^−^	595.1263	595.129	−4.5	C_26_H_28_O_16_	495, 479, 447, 425, 333, 300, 271	Quercetin-3-arabinoside-7-glucoside	273,676
10	3.824	[M−H]^−^	353.0856	353.0867	−3.1	C_16_H_18_O_9_	333, 316, 271, 191, 175	Chlorogenic acid	7520
11	3.876	[M−H]^−^	325.0911	325.0918	−2.2	C_15_H_18_O_8_	318, 305, 181, 146, 135	4-*O*-β-d-glucosy-l-4-coumarate	6416
12	3.932	[M−H]^−^	623.1583	623.1602	−3.0	C_28_H_32_O_16_	595, 480, 479, 463, 461, 301, 300	Isorhamnetin-3-*O*-neohespeidoside	3704
13	3.982	[M−H]^−^	495.0743	495.0768	−5.0	C_21_H_20_O_14_	387, 334, 333, 316, 305, 179, 137	Hibiscetin-3-*O*-glucoside	195,097
14	3.987	[M−H]^−^	741.1829	741.1866	−5.0	C_32_H_38_O_20_	611, 495, 387, 333, 300	Quercetin-3-glucosyl-(1->4)-xylosyl-(1->4)-Rhamnoside	13,845
15	3.993	[M−H]^−^	137.0242	137.0237	3.6	C_7_H_6_O_3_	—	Salicylic acid	1120
16	4.026	[M−H]^−^	479.0792	479.0819	−5.6	C_21_H_20_O_13_	447, 417, 223, 179, 61	Cannabiscitrin	759,151
17	4.067	[M−H]^−^	625.1361	625.1395	−5.4	C_27_H_30_O_17_	521, 480, 479, 463, 337, 300, 271	Myricetin-3-neohesperidoside	197,243
18	4.080	[M−H]^−^	289.0679	289.0708	−10.0	C_15_H_14_O_6_	271, 255, 210, 151	Cianidanol	855
19	4.101	[M−H]^−^	609.1406	609.1446	−6.6	C_27_H_30_O_16_	542, 480, 479, 463, 385, 300, 210	Rutin	614,274
	4.336	[M+H]^+^	611.1582	611.1602	−3.3	C_27_H_30_O_16_	531, 450, 447, 445, 439, 229	Rutin	959
20	4.120	[M−H]^−^	179.0348	179.0342	3.4	C_9_H_8_O_4_	167	Caffeic acid	181
21	4.233	[M−H]^−^	463.0839	463.087	−6.7	C_21_H_20_O_12_	381, 273, 315, 151	Hyperoside	2,443,396
22	4.503	[M−H]^−^	463.0839	463.087	−6.7	C_21_H_20_O_12_	461, 455, 301, 151	Isoquercetin	1,713,071
23	4.662	[M−H]^−^	163.0391	163.0393	−1.2	C_9_H_8_O_3_	139	Trans-p-coumaric acid	1168
24	4.679	[M−H]^−^	165.0546	165.0549	−1.8	C_9_H_10_O_3_	139	Ethylparaben	1965
25	4.854	[M−H]^−^	447.0907	447.0921	−3.1	C_21_H_20_O_11_	326, 318, 317, 195, 139	Astragalin	1953
26	4.893	[M−H]^−^	477.0999	477.1026	−5.7	C_22_H_22_O_12_	445, 429, 300, 271, 243, 242, 231	Isorhamnetin-3-glucoside	6568
27	4.928	[M−H]^−^	447.0913	447.0921	−1.8	C_21_H_20_O_11_	301, 178, 151	Isoorientin	303
28	5.114	[M−H]^−^	493.0577	493.0614	−7.5	C_21_H_18_O_14_	385, 339, 318, 243, 139	Hibifolin	3,417,545
29	5.171	[M−H]^−^	463.0845	463.087	−5.4	C_21_H_20_O_12_	461, 439, 319, 301, 243, 165	Quercetin 3′-*O*-β-d-glucoside	1,144,905
30	5.248	[M−H]^−^	447.0919	447.0921	−0.4	C_21_H_20_O_11_	385, 339, 323, 319, 300, 271, 137	Quercitrin	118
31	5.307	[M−H]^−^	187.0962	187.0966	−2.1	C_9_H_16_O_4_	—	Nonanedioic acid	985
32	5.396	[M−H]^−^	505.0951	505.0975	−4.8	C_23_H_22_O_13_	445, 339, 317, 316, 315, 146, 137	Quercetin-3-*O*-(6″-acetyl-glucoside)	60,696
33	5.468	[M−H]^−^	317.0271	317.0294	−7.3	C_15_H_10_O_8_	315, 178, 151, 109	Myricetin	1,193,716
34	5.672	[M−H]^−^	505.0934	505.0975	−8.1	C_23_H_22_O_13_	445, 323, 301, 300, 202, 121	Quercetin-3-(2″-acetyl-galactoside)	52,592
35	6.028	[M−H]^−^	489.105	489.1026	4.9	C_23_H_22_O_12_	409, 307	3-*O*-beta-d-Glucuronoside,etester-3,4′,5,7-tetrahydroxyflavone	68
36	6.382	[M−H]^−^	465.1732	465.1752	−4.3	C_23_H_30_O_10_	303, 259, 215	Ilexin II	1658
37	6.451	[M−H]^−^	201.1123	201.1122	0.5	C_10_H_18_O_4_	179	Sebacic acid	68
38	6.846	[M−H]^−^	593.1281	593.1287	−1.0	C_30_H_26_O_13_	545, 401, 313, 285, 129	Tribuloside	219
39	6.949	[M−H]^−^	301.0324	301.0345	−7.0	C_15_H_10_O_7_	249, 179, 151	Quercetin	1,500,688
40	7.794	[M−H]^−^	395.2022	395.2061	−9.9	C_21_H_32_O_7_	327, 215	Furonewguinone B	792
41	8.101	[M−H]^−^	269.0493	269.0447	17.1	C_15_H_10_O_5_	—	Genistein	38
42	8.435	[M−H]^−^	285.0388	285.0396	−2.8	C_15_H_10_O_6_	—	Kaempferol	2541
43	8.565	[M−H]^−^	315.049	315.0501	−3.5	C_16_H_12_O_7_	300, 151	Isorhamnetin	6714
44	8.751	[M−H]^−^	289.1433	289.1434	−0.3	C_17_H_22_O_4_	171, 130	6-Isobutyryl-5,7-dimethoxy-2,2-dimethyl benzopyran	378
45	8.831	[M−H]^−^	491.118	491.1182	−0.4	C_23_H_24_O_12_	401, 357, 313, 121	Yixingensin	357
46	9.363	[M−H]^−^	287.0933	287.0915	6.3	C_16_H_16_O_5_	—	Alkannin	112
47	12.557	[M−H]^−^	593.1258	593.1287	−4.9	C_30_H_26_O_13_	474, 397, 277, 239, 129, 112	Kaempferol-3-glucoside-2″-p-coumaroyl	5171
48	13.537	[M−H]^−^	255.2316	255.2316	0.0	C_16_H_32_O_2_	145	Palmitic acid	1147
49	13.880	[M−H]^−^	361.1978	361.2007	−8.0	C_21_H_30_O_5_	293, 255, 130	Enaimeone B	1448
50	14.022	[M−H]^−^	277.144	277.1434	2.2	C_16_H_22_O_4_	255, 179	Diisobutyl phthalate	539
51	14.713	[M−H]^−^	297.1505	297.1485	6.7	C_19_H_22_O_3_	255, 183, 149	Aurapten	7267
52	14.798	[M−H]^−^	225.2195	225.2211	−7.1	C_15_H_30_O	101	2-Pentadecanone	61
53	15.103	[M−H]^−^	279.2301	279.2316	−5.4	C_18_H_32_O_2_	208, 150, 100	9,12-Octadecadienoic acid	4641
54	16.199	[M−H]^−^	389.2709	389.2682	6.9	C_24_H_38_O_4_	313, 267	Diisooctyl phthalate	27
55	3.961	[M+H]^+^	525.229	525.2325	−6.7	C_26_H_36_O_11_	520, 213	Neocaesalpin L	229
56	4.370	[M+H]^+^	582.1758	582.1813	−9.4	C_26_H_28_O_14_	428, 425, 423, 417, 229	3,4,5-Trihydroxy-6-[7-hydroxy-2-(3-hydroxy-4-methoxyphenyl)-6,8-dimethoxy-4-oxochromen-5-yl]oxyoxan-2-yl]methylacetate	119
57	4.609	[M+H]^+^	563.236	563.2348	2.1	C_27_H_34_N_5_O_7_	495, 494, 492, 489, 483, 481	1-[2-(2-acetylammonium)-3-phenyl propionyl-pyrrolidine-2-carboxylic acid	22
58	4.668	[M+H]^+^	505.2594	505.2637	−8.5	C_24_H_40_O_11_	479, 477, 470, 271, 163	leeaoside	323
59	8.178	[M+H]^+^	277.2848	277.2862	−5.0	C_18_H_38_	266, 261, 244, 213	*n*-Octadecane	207
60	12.800	[M+H]^+^	522.114	522.1183	−8.2	C_30_H_16_O_8_	460, 454, 453, 437, 415, 299, 119	Hypericin	172
61	12.801	[M+H]^+^	415.2805	415.2838	−7.9	C_26_H_38_O_4_	303, 299, 247, 135, 119	Hyperpapuanone	20,517
62	15.393	[M+H]^+^	457.3708	457.3669	8.5	C_30_H_48_O_3_	413, 371, 369, 355	Ursolic acid	1389
63	16.636	[M+H]^+^	311.0865	311.0891	−8.4	C_16_H_16_O_5_	170, 140	(2S)-5,3′4′-trihydroxy-7-methoxyflavane	211

Note: ”—” means no Fragments.

**Table 4:** Identified chemical constituents from RFF by UPLC-QTOF-MS in positive and negative ion mode.

NO.	RT/min	Precursor Ion	Meas.*m*/*z*	*m*/*z*	Error/ppm	Formula	Fragments	Identification	Response
1	0.614	[M−H]^−^	289.0685	289.0708	−8.0	C_15_H_14_O_6_	191, 133, 128	Cianidanol	583
2	0.658	[M−H]^−^	503.1603	503.1602	0.2	C_18_H_32_O_16_	379, 377, 321, 215, 195, 161, 133	Gentianose	506
	13.215	[M+H]^+^	522.2048	522.2048	0.0	C_18_H_32_O_16_	500, 478, 457, 384, 313, 188, 122	Gentianose	418
3	0.668	[M−H]^−^	266.0871	266.0887	−6.0	C_10_H_13_N_5_O_4_	164, 150	Adenosine	6287
4	1.493	[M−H]^−^	285.0588	285.0606	−6.3	C_12_H_14_O_8_	195	Uralenneoside	110
5	2.697	[M−H]^−^	303.0843	303.0864	−6.9	C_16_H_16_O_6_	253, 249, 232, 164, 147, 94	3-Methoxypyrocatechol	152
6	3.548	[M−H]^−^	153.0178	153.0186	−5.2	C_7_H_6_O_4_	—	Protocatechuic acid	62,326
7	3.549	[M−H]^−^	109.0290	109.0288	1.8	C_6_H_6_O_2_	—	Hydroquinone	997
8	3.686	[M−H]^−^	369.0851	369.0816	9.5	C_16_H_18_O_10_	316, 291, 287, 203, 153	Fraxin	43
9	3.795	[M−H]^−^	479.0791	479.0819	−5.8	C_21_H_20_O_13_	463, 339, 301, 271, 269, 190	Myricetin-3-*O*-β-d-glucoside	214,470
10	3.797	[M−H]^−^	595.1255	595.1290	−5.9	C_26_H_28_O_16_	463, 439, 301, 255, 243	Quercetin-3-arabinoside-7-glucoside	308,061
	16.912	[M+H]^+^	597.1513	597.1556	−7.2	C_26_H_28_O_16_	539, 496, 393, 355, 323, 263	Quercetin-3-arabinoside-7-glucoside	849
11	3.821	[M−H]^−^	353.0850	353.0867	−4.8	C_16_H_18_O_9_	290, 200, 128, 161	Chlorogenic acid	6601
12	3.931	[M−H]^−^	623.1574	623.1602	−4.5	C_28_H_32_O_16_	595, 479, 314, 281, 271, 255	Isorhamnetin-3-*O*-neohespeidoside	9866
13	3.932	[M−H]^−^	179.0346	179.0342	2.2	C_9_H_8_O_4_	147	Caffeic acid	2735
14	3.982	[M−H]^−^	495.0747	495.0768	−4.2	C_21_H_20_O_14_	464, 463, 333, 303, 179	Hibiscetin-3-*O*-glucoside	10,639
15	3.984	[M−H]^−^	741.1816	741.1866	−6.7	C_32_H_38_O_20_	612, 611, 495, 461, 323, 316, 300, 287, 151, 137	Quercetin-3-glucosyl-(1->4)-xylosyl-(1->4)-rhamnoside	15,593
16	3.988	[M−H]^−^	137.0233	137.0237	−2.9	C_7_H_6_O_3_	124	Salicylic acid	5443
17	4.026	[M−H]^−^	479.0780	479.0819	−8.1	C_21_H_20_O_13_	477, 318, 316, 291, 287, 179, 201	Cannabiscitrin	354,843
18	4.110	[M−H]^−^	609.1393	609.1446	−8.7	C_27_H_30_O_16_	441, 300, 255, 243, 151	Rutin	1,226,910
	5.367	[M+H]^+^	628.1877	628.1867	1.6	C_27_H_30_O_16_	551, 487, 481, 367, 352, 319, 254	Rutin	763
19	4.231	[M−H]^−^	463.0832	463.0870	−8.2	C_21_H_20_O_12_	461, 303, 179	Hyperoside	776,080
20	4.307	[M−H]^−^	625.1360	625.1395	−5.6	C_27_H_30_O_17_	565, 521, 477, 466, 319, 316, 303	Myricetin-3-neohesperidoside	153,458
	12.994	[M+H]^+^	649.1419	649.1371	7.4	C_27_H_30_O_17_	640, 556, 494, 337, 188, 184, 104	Myricetin-3-neohesperidoside	1343
21	4.498	[M−H]^−^	463.0828	463.0870	−9.1	C_21_H_20_O_12_	455, 301, 151	Isoquercetin	2,318,366
	1.090	[M+H]^+^	482.1275	482.1275	0.0	C_21_H_20_O_12_	423, 330, 314, 296, 274, 203, 130	Isoquercetin	523
22	4.561	[M−H]^−^	593.1506	593.1497	1.5	C_27_H_30_O_15_	493, 479, 318, 317, 139	Aempferol-3-*O*-rutinoside	179
23	4.673	[M−H]^−^	165.0541	165.0549	−4.8	C_9_H_10_O_3_	139	Ethylparaben	7244
24	4.691	[M−H]^−^	167.0345	167.0342	1.8	C_8_H_8_O_4_	165	Vanillic acid	846
25	4.888	[M−H]^−^	477.0996	477.1026	−6.3	C_22_H_22_O_12_	345, 323, 300, 271, 245, 243, 164, 231, 169	Isorhamnetin-3-glucoside	4277
26	4.931	[M−H]^−^	447.0938	447.0921	3.8	C_21_H_20_O_11_	315, 301, 240, 151	Isoorientin	271
27	5.114	[M−H]^−^	493.0565	493.0614	−9.9	C_21_H_18_O_14_	343, 243, 380, 339, 193	Hibifolin	5,596,770
	1.624	[M+H]^+^	517.0566	517.0588	−4.3	C_21_H_18_O_14_	365, 266, 210, 203, 140, 136	Hibifolin	89
28	5.175	[M−H]^−^	463.0826	463.0870	−9.5	C_21_H_20_O_12_	323, 301, 178, 151	Quercetin 3′-*O*-β-d-glucoside	530,689
29	5.242	[M−H]^−^	447.0896	447.0921	−5.6	C_21_H_20_O_11_	407, 385, 317, 285, 249, 150, 137	Quercitrin	64
30	5.307	[M−H]^−^	187.0962	187.0966	−2.1	C_9_H_16_O_4_	179, 151	Nonanedioicacid	1519
31	5.396	[M−H]^−^	505.0950	505.0975	−4.9	C_23_H_22_O_13_	406, 300, 137, 61	Quercetin-3-*O*-(6″-acetyl-glucoside)	34,203
	1.689	[M+H]^+^	524.1377	524.1396	−3.6	C_23_H_22_O_13_	258, 176, 182, 165, 144, 91	Quercetin-3-*O*-(6″-acetyl-glucoside)	1155
32	5.462	[M−H]^−^	317.0267	317.0294	−8.5	C_15_H_10_O_8_	300, 178, 62	Myricetin	1,338,137
33	5.665	[M−H]^−^	505.0934	505.0975	−8.1	C_23_H_22_O_13_	445, 377, 301, 300, 121	Quercetin-3-(2″-acetyl-galactoside)	24,390
34	6.141	[M−H]^−^	201.1136	201.1122	7.0	C_10_H_18_O_4_	—	Sebacic acid	202
35	6.763	[M−H]^−^	287.0561	287.0552	3.1	C_15_H_12_O_6_	137	Steppogenin	610
36	6.838	[M−H]^−^	593.1276	593.1287	−1.9	C_30_H_26_O_13_	549, 505, 493, 319, 245, 167	Tribuloside	181
37	6.947	[M−H]^−^	301.0320	301.0345	−8.3	C_15_H_10_O_7_	253, 271, 179, 151	Quercetin	4,286,867
38	8.424	[M−H]^−^	285.0382	285.0396	−4.9	C_15_H_10_O_6_	255, 145	Kaempferol	11,321
39	8.555	[M−H]^−^	315.0485	315.0501	−5.1	C_16_H_12_O_7_	300, 239, 151	Isorhamnetin	9328
40	8.738	[M−H]^−^	289.1431	289.1434	−1.0	C_17_H_22_O_4_	—	6-iso butyryl-5, 7-dimethoxy-2, 2-dimethyl-benzopyran	1277
41	8.823	[M−H]^−^	491.1168	491.1182	−2.9	C_23_H_24_O_12_	401, 393, 357, 313, 121	Yixingensin	319
42	12.047	[M−H]^−^	431.0931	431.0972	−9.5	C_21_H_20_O_10_	363, 295, 195	Vitexin	46
43	12.555	[M-H]^−^	593.1276	593.1287	−1.9	C_30_H_26_O_13_	474, 397, 386, 277, 196, 113	Kaempferol-3-glucoside-2″-p-coumaroyl	1854
44	13.040	[M−H]^−^	439.3537	439.3564	−6.1	C_30_H_48_O_2_	367, 313, 266, 149	Roburic acid	175
	12.897	[M+H]^+^	458.4014	458.3985	6.3	C_30_H_48_O_2_	444, 424, 390, 337, 335, 132, 124	Roburic acid	473
45	13.529	[M−H]^−^	255.2336	255.2316	7.8	C_16_H_32_O_2_	—	Palmitic acid	188
46	13.883	[M−H]^−^	361.1972	361.2007	−9.7	C_21_H_30_O_5_	327, 293	Enaimeone B	5252
47	14.160	[M−H]^−^	283.2634	283.2628	2.1	C_18_H_36_O_2_	145, 125, 117	Ethyl palmitate	76
	2.738	[M+H]^+^	285.2761	285.2784	−8.1	C_18_H_36_O_2_	153, 136	Ethyl palmitate	853
48	14.405	[M−H]^−^	345.2632	345.2631	0.3	C_19_H_38_O_5_	342, 299, 267, 150	1, 1, 3, 3-tetrabutoxy-2-propanone	237
49	14.716	[M−H]^−^	297.1509	297.1485	8.1	C_19_H_22_O_3_	265, 150, 100	Aurapten	2788
50	14.795	[M−H]^−^	225.2222	225.2211	4.9	C_15_H_30_O	183	2-Pentadecanone	105
51	14.795	[M−H]^−^	371.1506	371.1488	4.8	C_21_H_24_O_6_	273, 271, 265, 225, 183	(-)-Arctigenin	1025
	3.613	[M+H]^+^	395.1451	395.1464	−3.3	C_21_H_24_O_6_	391, 367, 305, 300, 229, 192, 156	(-)-Arctigenin	233
52	15.101	[M−H]^−^	279.2299	279.2316	−6.1	C_18_H_32_O_2_	255, 117	9, 12-*O*-ctadecadienoic acid	5429
	6.965	[M+H]^+^	303.2272	303.2292	−6.6	C_18_H_32_O_2_	233, 153	9, 12-*O*-ctadecadienoic-acid	147,858
53	15.201	[M−H]^−^	455.3493	455.3513	−4.4	C_30_H_48_O_3_	385, 265, 183, 150, 121	Ursolic acid	1416
	15.397	[M+H]^+^	457.3698	457.3669	6.3	C_30_H_48_O_3_	413, 369, 347, 270, 130	Ursolic acid	2232
54	16.511	[M−H]^−^	603.3875	603.3882	−1.2	C_35_H_56_O_8_	381, 356, 355, 309, 307, 119	Ziyuglycoside II	219
55	0.951	[M+H]^+^	397.2181	397.2217	−9.1	C_21_H_32_O_7_	383, 266, 248, 203, 191	Furonewguinone B	2102
56	0.983	[M+H]^+^	489.1688	489.1728	−8.2	C_23_H_30_O_10_	381, 365, 360, 203	Ilexin II	974
57	1.109	[M+H]^+^	522.1223	522.1183	7.7	C_30_H_16_O_8_	423, 330, 314, 296, 274, 256	Hypericin	804
58	2.459	[M+H]^+^	263.2720	263.2706	5.3	C_17_H_36_	120	*n*-Heptadecane	156
59	2.871	[M+H]^+^	345.2080	345.2058	6.4	C_21_H_28_O_4_	328, 323, 298, 166, 146, 132, 120	Ialibinone B	4759
60	3.507	[M+H]^+^	311.3695	311.3666	9.3	C_22_H_46_	298, 280	Docosane	5309
61	4.405	[M+H]^+^	960.5183	960.5146	3.9	C_47_H_74_O_19_	814, 792, 487, 397, 319, 277, 114	Deslanoside	5645
62	4.623	[M+H]^+^	255.3029	255.3042	−5.1	C_18_H_38_	152, 120	*n*-Octadecane	2184
63	4.856	[M+H]^+^	341.3401	341.3408	−2.1	C_22_H_44_O_2_	287, 262	Docosanoic acid	68
64	5.814	[M+H]^+^	612.1946	612.1918	4.6	C_27_H_30_O_15_	600, 580, 581, 579, 277, 233	Glucosylvitexin	2889
65	6.252	[M+H]^+^	554.4688	554.4711	−4.1	C_40_H_56_	473, 471, 303, 287, 247	beta-Carotene	79
66	6.285	[M+H]^+^	297.3528	297.3510	6.1	C_21_H_44_	262, 222	Heneicosane	307
67	7.825	[M+H]^+^	687.6081	687.6036	6.5	C_46_H_80_O_2_	—	Alpha-Amyrin palmitate	666
68	8.324	[M+H]^+^	391.2804	391.2838	−8.7	C_24_H_38_O_4_	375, 354, 353, 348, 195	Diisooctyl phthalate	382
69	8.701	[M+H]^+^	612.1686	612.1680	1.0	C_27_H_31_O_16_	610, 496, 351, 325, 249, 147	Enocyanin	388
70	9.048	[M+H]^+^	394.2550	394.2584	−8.6	C_22_H_32_O_5_	334, 277	Enaimeone C	242
71	9.101	[M+H]^+^	553.3833	553.3879	−8.3	C_35_H_52_O_5_	427, 351, 311	Furohyperforin	695
72	11.743	[M+H]^+^	430.4040	430.4036	0.9	C_29_H_48_O	421, 411, 378, 310, 307	α-Spinasterol	811
73	12.904	[M+H]^+^	657.3733	657.3753	−3.0	C_39_H_54_O_7_	561, 554, 553, 303, 196	3-*O*-trans-p-coumaroyltormentic acid	292
74	12.909	[M+H]^+^	651.4093	651.4092	0.2	C_36_H_58_O_10_	599, 588, 453, 344, 335, 200, 124	Pedunculoside	254
75	13.263	[M+H]^+^	547.2141	547.2145	−0.7	C_26_H_36_O_11_	543, 542, 520, 483, 313, 184	Neocaesalpin L	2487
76	13.312	[M+H]^+^	625.1723	625.1758	−5.6	C_28_H_32_O_16_	616, 532, 437, 393, 372, 366, 307	Narcissoside	9633
77	13.332	[M+H]^+^	339.3947	339.3978	−9.1	C_24_H_50_	299, 188	Tetracosane	4602
78	13.469	[M+H]^+^	541.2522	541.2528	−1.1	C_27_H_34_N_5_O_7_	463, 391, 358, 339, 342, 283, 277	1-[2-(2-acetyl ammonium)-3-phenylpropionate-pyrrolidine-2-carboxylic acid	6476
79	13.602	[M+H]^+^	661.1721	661.1734	−2.0	C_29_H_34_O_16_	569, 310, 309, 284, 133, 89	Quercetin-3, 3′-dimethyl ether-7-rutinoside	16,922
80	13.729	[M+H]^+^	557.3544	557.3594	−9.0	C_35_H_50_O_4_	544, 522, 485, 480, 339, 184, 104	Pyrano-[7, 28-b]-hyperforin	386
81	13.824	[M+H]^+^	757.2179	757.2178	0.1	C_33_H_40_O_20_	699, 684, 640, 597, 330, 305, 118	Kaempferol-3-sophoroside-7-rhamnoside	361
82	14.208	[M+H]^+^	527.2478	527.2457	4.0	C_24_H_40_O_11_	395, 297, 279, 278	leeaoside	450,363
83	14.327	[M+H]^+^	696.6235	696.6274	−5.6	C_46_H_78_O_3_	541, 349, 344, 226	11α, 12α-Oxidotaraxerol palMitate	767
84	15.501	[M+H]^+^	701.1445	701.1497	−7.4	C_36_H_28_O_15_	690, 589, 567, 545, 501, 283	Theaflavin gallate	381
85	15.513	[M+H]^+^	679.1591	679.1653	−9.1	C_34_H_30_O_15_	668, 593, 567, 545, 523, 457, 413	3, 4, 5-Tricaffeoylquinic acid	1207
86	16.462	[M+H]^+^	575.3692	575.3699	−1.2	C_35_H_52_O_5_	559, 437, 227	8-Hydroxyhyperforin-8, 1-hemiacetal	437
87	16.644	[M+H]^+^	594.4730	594.4717	2.2	C_35_H_60_O_6_	546, 487, 145	Daucosterol	372

Note: ”—” means no Fragments.

**Table 5:** Factors and levels of response surface analysis test for the total flavonoid extraction process of fermented HML.

Factors	Coded Symbols	Levels		
		−1	0	1
Material–liquid ratio (g/mL)	A	1:35	1:40	1:45
Extraction time (h)	B	1.5	2	2.5
Ethanol concentration (%)	C	50	60	70
Extraction temperature (°C)	D	70	80	90

## Data Availability

The data presented in this study are available in article and supplementary materials.

## Supplementary Materials

The following supporting information can be downloaded at: https://www.mdpi.com/article/10.3390/molecules29051046/s1, Figure S1: Rutin standard curve determination of total flavonoid content.
