# Supplementary material for: Effects of Rhizopus-arrhizus-31-Assisted Pretreatment on the Extraction and Bioactivity of Total Flavonoids from Hibiscus manihot L
Source: Molecules. 2024 Feb 28;29(5):1046. doi: 10.3390/molecules29051046 (PMC10934683; doi:10.3390/molecules29051046)
Supplement: Supplementary file 1 [file molecules-29-01046-s001.zip › molecules-2818941-supplementary.pdf]

Supplementary Materials

Total flavonoids content in HML was measured using the method described in the 2020 China Pharmacopoeia. We prepared a standard solution by weighing 20 mg of rutin, dissolving it in 30% ethanol, and heating it in a water bath. The solution was then diluted to a volume of 100 mL to obtain a 0.2 mg/mL standard solution. For calibration, 1, 2, 3, 4, 5, and 6 mL aliquots of the standard solution were transferred to 25 mL volumetric flasks. Each flask was then filled to the 6 mL mark with 30% ethanol, followed by the sequential addition of 1 mL of 5% sodium nitrite solution, 1 mL of 10% aluminum nitrate solution, and 10 mL of 4% sodium hydroxide solution. After allowing the solutions to react for 6 minutes and 15 minutes, respectively, the flasks were filled to the mark with 30% ethanol and mixed thoroughly. A blank, prepared without sodium hydroxide, was also included. The absorbance of each solution was measured at 500 nm using a spectrophotometer. A standard curve was then constructed by plotting the rutin concentration (C, mg/mL) on the x-axis and the corresponding absorbance on the y-axis. The linear regression equation of the standard curve was $y=9.17143x+0.0052$. The correlation coefficient $R^2=0.9999$.

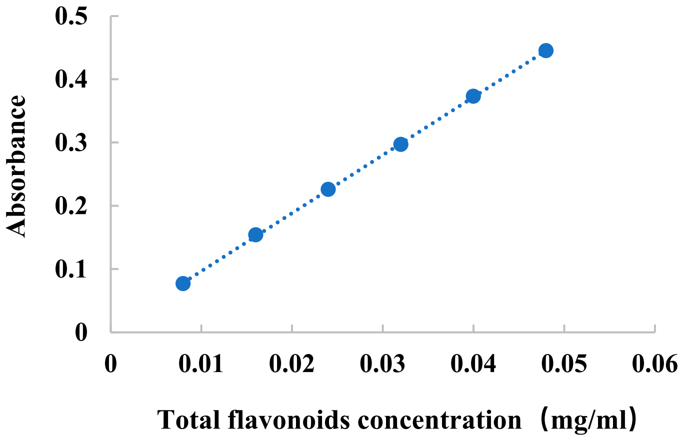

Figure S1. Rutin standard curve determination of total flavonoids content.
